# A Case Report of Infective Endocarditis With Icterus

**DOI:** 10.1002/ccr3.71370

**Published:** 2025-10-26

**Authors:** Ramtin Rezaie Kalantari, AmirAbas Sadrian, Torkan Banaee Rezaeie, Masoud Mortezazadeh, Dalip Kumar, Michael Acidri, Abbas Mofidi

**Affiliations:** ^1^ Department of General Surgery, School of Medicine Jundishapur University of Medical Sciences Ahvaz Iran; ^2^ Department of Internal Medicine, School of Medicine Jundishapur University of Medical Sciences Ahvaz Iran; ^3^ Department of Hematology‐Oncology, Cancer Institute, Imam Khomeini Hospital Complex Tehran University of Medical Sciences (TUMS) Tehran Iran; ^4^ Trauma Network–Southend Hospital, Mid and South Essex NHS Foundation Trust Southend University Hospital Southend UK; ^5^ Emergency Department, Mid and South Essex NHS Foundation Trust Southend University Hospital Southend UK; ^6^ Emergency Department ST3, Mid and South Essex NHS Foundation Trust Southend University Hospital Southend UK

**Keywords:** congestive hepatopathy, culture‐negative endocarditis, icterus, infective endocarditis, mitral valve repair, mitral valve vegetation

## Abstract

Infective endocarditis, a severe condition with high morbidity and mortality, requires timely diagnosis and treatment. We present a case of a 49‐year‐old male with a 3‐day history of icterus, abdominal pain, loose stool, and high‐grade fever. He was unwell for 3 days prior to presenting. Despite normal cardiac and abdominal examinations, transthoracic echocardiography revealed a large mitral valve mass with severe regurgitation, necessitating emergency surgery. Successful mitral valve repair and vegetation removal led to full recovery. This case underscores the importance of early echocardiography in patients with systemic symptoms such as icterus, abdominal pain, and fever to promptly identify infective endocarditis and prevent complications.

## 1 Introduction

Infective endocarditis is an inflammation of the inner lining of the heart caused by microbial pathogens, particularly bacterial infections, and affects various systems in the body. Early diagnosis and heart surgery can help reduce the risk of death [1]. Infective endocarditis is more common in patients with a history of heart surgery, people with prosthetic heart valves, venous catheters, pacemakers, and internal cardiac defibrillators. In addition, conditions such as tooth decay, periodontitis, injections with a contaminated syringe, recent invasive procedures such as dental treatments and tattoos, and intestinal infections such as inflammatory bowel disease can cause bacteria to enter the bloodstream and trigger infective endocarditis. Common symptoms include hyperpyrexia, nocturnal hyperhidrosis, dyspnea, dermatitis, angina pectoris, tachycardia, hematuria, anorexia, weight loss, and positive blood cultures, although negative blood cultures occur in 2%–20% of cases. Infective endocarditis often results in damage to the heart valves, which can lead to heart failure associated with pulmonary hypertension and liver dysfunction. Bilirubin, a breakdown product of heme, is included in liver function testing and elevated levels (hyperbilirubinemia) or jaundice can result from liver or bile duct dysfunction, which may occur as a complication of bacterial infections, especially in the setting of sepsis [2]. We report here a case of infective mitral valve endocarditis with yellowing of the eyes, abdominal pain, muscle pain, and negative blood culture.

## 2 Case History

A 49‐year‐old healthy male with no history of cardiovascular disease presented to a general medicine clinic in Ahwaz in July 2020 with mild and occasional fever for 10 days, alongside a 3‐day history of eye‐yellowing, abdominal pain in the epigastric region, diarrhea, nausea and vomiting, and muscle pain. The patient was temporarily admitted to the emergency department and developed a high fever (40°C) and restlessness the same night, after which he was admitted directly to the intensive care unit due to high‐grade fever and restlessness, suggestive of systemic inflammatory response or early septic shock which was managed with antipyretics and intravenous fluids.

### 2.1 Physical Examination

- No rash, skin lesions, or itching were observed.
- There was no swelling of the cervical, axillary, or inguinal lymph nodes, nor was there a thyroid disorder.
- The heart sounds were reported as normal and without any murmurs by the internal medicine resident.
- Chest examination was normal.
- Abdominal examination showed no tenderness, rigidity, ascites, or enlargement of the limbs or presence of masses.

The patient had no history of tattoos or infected injections and had only a few caries, but no invasive procedures had been performed on him. The patient had no history of underlying diseases such as diabetes and hypertension. The patient stated that they had taken unspecified herbal medicines (no additional details) a few days before the onset of symptoms.

### 2.2 Differential Diagnosis, Investigations, and Treatment

Ultrasound examination revealed no intraperitoneal collection or free fluid in the pelvis. In addition, the patient's COVID‐19 PCR test result was negative twice. The results of several blood cultures and troponin I of the patient during hospitalization were also negative. Fungal blood cultures were sent during hospitalization and returned negative, ruling out fungal etiology.

Preliminary test results are presented in Table 1. Urine analysis results were also normal (Table 2). CBC was checked on a daily basis, and anemia, decreased Hb level (10.9 g/dL) as well as decreased PLT count (105 × 10^3^ μL) were observed. Serum electrolytes (decreased sodium and potassium levels) and liver function tests (AST: 60 IU/L) were abnormal and bilirubin levels were high (TBil: 3.0 mg/dL).

**TABLE 1:** The patient's preliminary laboratory test results.

Variable	Result	Unit	Ref range
**Hematology**			
WBC	6.2	×10^3^/μL	4.4–11.2
RBC	3.72 L	×10^6^/μL	4.5–5.9
Hb	10.9 L	g/dL	13–18
HCT	41.2 L	%	40–52
MCV	90.35	fL	80–96
MCH	27.41	Pg	27.5–33.2
MCHC	30.34 L	g/dL	31.5–35.5
PLT	105 L	×10^3^/μL	150–450
ESR	50	mm/h	0–15
RDW	15.7	%	11–16
INR	1.3	—	
**Blood biochemistry**			
Total bilirubin	3.0 H	mg/dL	0.1–1.2
AST (S.G.O.T.)	60 H	IU/L	0–33
ALT (S.G.P.T.)	39	U/L	0–40
Alkaline phosphatase	214	U/L	98–279
Creatine phosphokinase	114	U/L	39–308
Uric acid	4.6	mg/dL	3.6–7.7
BUN	17	mg/dL	10–55
Creatinine	1.5	mg/dL	0.6–1.5
Cholesterol	185	mg/dL	< 200
TG	139	mg/dL	< 150
Na	132 L	mg/dL	135–145
K	4.0 L	mg/dL	3.5–5.1
FBS	92	mg/dL	160–115
**Serology**			
CRP	118.6	mg/L	< 5
**Immunoassay**			
HEV ab	Negative		
HAV ab	Negative		
HBs Ag	Negative		
HBc ab	Negative		
HCV ab	Negative		
HBC ab	Negative		
HEV IgM	Negative		
Anti HIV	Negative		

**TABLE 2:** Urine analysis results.

Macroscopic	Result	Microscopic	Results
Color	Deep yellow	WBC	7–9 cell/LPF
App	Clear	RBC	5–7 cell/LPF
pH	5	Epithelial	0–1
SG	1012	Bacteria	Few
Albumin	Trace	Crystal	Amorphous urate
Ketones	Negative		
Glucose	Negative		
Bill	Negative		
Urobilinogen	Negative		
Nitrite	Negative		
Hemo	Negative		

Acute hepatitis and cholangitis were suspected as differential diagnosis beside systemic symptoms suggestive of early sepsis, leading to intravenous treatment with broad‐spectrum antibiotics such as cephalexin, ampicillin, ciprofloxacin, and clindamycin. During hospitalization, the total bilirubin level gradually decreased (2.3 mg/dL on the second day, 1.6 mg/dL on the third day, and 1.2 mg/dL on the fourth day of hospitalization).

### 2.3 Further Course and Definitive Diagnosis

**FIGURE 1:**
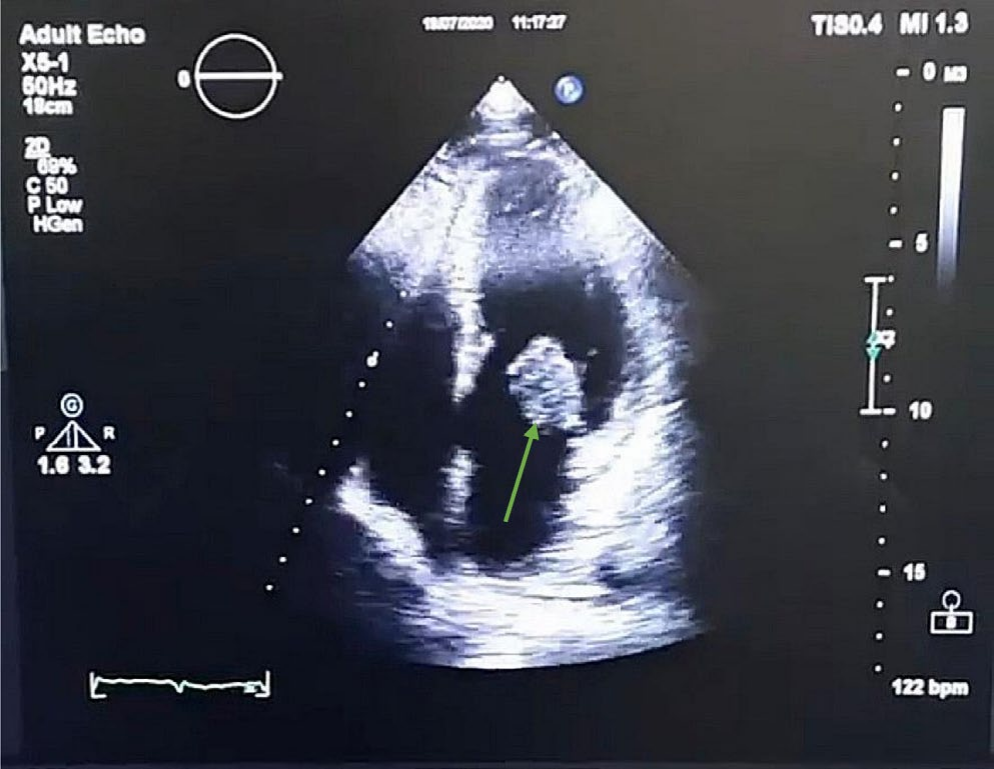
Echocardiographic image before the patient's heart surgery. A vegetation of about 2.5 cm was observed on the posterior leaflet of the mitral valve, which is marked by green arrow.

The patient's fever persisted after 4 days of antibiotic therapy and he went into septic shock. After ruling out other possible differential diagnoses for fever of unknown origin (FUO) and persistent fever above 38°C in spite of management, infective endocarditis was suspected. He underwent TTE echocardiography (Figure 1).

### 2.4 TTE Findings

- Severe mitral regurgitation (MR) was observed.
- A large, hypermobile mass (2.5 × 2.2 cm) was detected on the mitral valve attached to the posterior leaflet (PMVL), confirming the presence of vegetation.
- Mild pulmonary arterial hypertension (PAH) (PAP: 41 mmHg) was present.
- LV systolic function was normal (EF: 55%).
- Normal RV size (2.6 cm), normal RV systolic function (TAPSE: 20 mm).
- Normal atrial size and no mitral stenosis (MS).

The patient was therefore diagnosed with infective endocarditis with mitral vegetation and severe MR. An infectious disease specialist was consulted immediately after echocardiography confirmed mitral valve vegetation and severe regurgitation, guiding the continuation of broad‐spectrum antibiotics and postoperative management. Emergency angiography prior to cardiac surgery did not reveal coronary artery occlusion. The patient underwent immediate open‐heart surgery, vegetectomy (removal of vegetation), and the mitral valve was repaired. The surgical procedure involved excision of a 2.5 × 2.2 cm vegetation from the posterior mitral valve leaflet, followed by mitral valve repair using an annuloplasty ring. No prosthetic valve was implanted, preserving the native valve. The operation was completely successful and without complications.

### 2.5 Conclusion and Results (Outcome and Follow‐Up)

Postoperative echocardiography showed no visible vegetation on the MV. The size of the LV, RV, and atrium as well as the systolic function of the left ventricle was also normal (EF: 55%). Only mild thickening of the MV leaflets was observed, which was due to MV dysfunction. Two days after surgery, bacteriological tests and blood cultures were negative as no bacterial growth or fungal growth was observed. Non‐bacterial thrombotic endocarditis was considered but deemed unlikely due to the large, hypermobile vegetation and clinical evidence of sepsis. The PLT count was increased (172 × 10^3^/μL), but the patient still had fever, and antibiotic treatment was continued. On the fifth day after surgery, the PLT count continued to increase (307 × 10^3^/μL), but the fever decreased. On the seventh day after surgery and completion of antibiotic therapy, the patient was finally discharged in good general condition. The patient was scheduled for outpatient follow‐up at 1, 3, 6, and 12 months post‐discharge, including clinical evaluation, transthoracic echocardiography, and laboratory tests (complete blood count, liver function tests, inflammatory markers).

## 3 Discussion

Infective endocarditis with severe MR is usually a rapid indication for cardiac surgery. We report a rare presentation of infective endocarditis (IE) characterized by significant icterus, abdominal pain, and muscle aches, initially misdiagnosed as acute hepatitis, despite subsequent progression to septic shock. The absence of cardiac murmurs on initial examination posed a diagnostic challenge, as it delayed suspicion of infective endocarditis until persistent fever and echocardiographic findings confirmed the diagnosis. This case underscores the diagnostic challenge posed by atypical IE presentations, particularly the value of early echocardiography in patients with systemic inflammatory response syndrome or sepsis of unknown origin.

The icterus and elevated total bilirubin (3.0 mg/dL) were prominent features. Hyperbilirubinemia in IE is often multifactorial, stemming from either the systemic inflammatory response of sepsis or, as strongly suggested in this case, congestive hepatopathy secondary to acute, severe heart failure. Acute MR leads to rapidly increased left atrial and pulmonary venous pressure, causing pulmonary hypertension and right ventricular strain (evidenced by mild pulmonary arterial hypertension [PAP: 41 mmHg] in our patient). This ultimately results in massive hepatic congestion and subsequent liver dysfunction (elevated AST, ALT, and bilirubin). This mechanism aligns with other severe case reports of IE‐related liver dysfunction.

In China, Lin et al. [3] reported a case of infective endocarditis with multiple mitral valve vegetations, severe MR, severe hyperbilirubinemia (200 μmol/L), and positive bacterial blood culture ( *Streptococcus sanguinis* ), which was successfully improved by mitral valve repair. Echocardiography showed vegetation on the anterior and posterior leaflets of the mitral valve with mild pulmonary hypertension, and mitral valve repair was successfully performed.

The management of severe IE requires timely intervention. Current guidelines strongly recommend early surgical intervention for IE complicated by severe valve regurgitation, uncontrolled infection, and large vegetations (as seen in our case), particularly to mitigate the risk of systemic embolization and persistent sepsis [4, 5].

Impaired liver function tests, including high AST, ALT, and bilirubin levels, are often seen in patients with acute heart failure, and only in 29% of cases all liver function parameters are within the normal range [6]. Studies have also shown that preoperative hepatic dysfunction defined by icterus and high total bilirubin level is independently associated with in‐hospital mortality and morbidity in patients with infective endocarditis and can be used as a marker and a new strategy for risk assessment [7, 8]. Although high bilirubin is recognized as a risk factor in open‐heart surgery, emergency surgical intervention is the only option to treat and rescue the patient. Mitral valve repair is also associated with a better outcome than valve replacement, especially in younger patients, including better survival and quality of life [9]. In our report, the patient's bilirubin level decreased during hospitalization and the patient's operation was successful and without complications.

In various studies, bacterial agents, especially *Staphylococcus* and *Streptococcus* species, have been identified as the main microorganisms for infective endocarditis.

Our patient also exemplifies culture‐negative IE, which accounts for up to 20% of cases. The presence of icterus despite negative blood cultures may be attributed to systemic inflammation and sepsis‐induced liver dysfunction, potentially exacerbated by hemolysis from mitral regurgitation or subclinical bacterial seeding not detected by standard cultures. While prior antibiotic therapy for suspected hepatitis may have sterilized the blood cultures, the potential for fastidious organisms (e.g., *Bartonella*, *Coxiella*) or fungal etiologies must also be considered, though a definitive etiological agent was not identified. Given the patient's dramatic response to vegetectomy and continued broad‐spectrum antibiotic therapy, the surgical removal of the infectious burden proved curative [10, 11].

Therefore, the use of automatic blood culture and PCR techniques can help identify resistant species that are difficult to detect. In addition, the diagnosis of infective endocarditis in patients with symptoms of icterus and persistent fever without a history of infected injection and any recent invasive intervention can be difficult. Therefore, the suspicion of endocarditis in patients with unexplained fever or any symptoms of systemic disease should be considered. Additionally, because high bilirubin is a risk factor for death from severe sepsis in patients with infective endocarditis, echocardiography is recommended for early diagnosis and management in patients with these symptoms due to its high accuracy and sensitivity for the detection of vegetations [12, 13], and being quick to perform regardless of the risk factors for infective endocarditis.

It can therefore be concluded that jaundice, abdominal pain and negative blood cultures are rare symptoms of infective left ventricular endocarditis and that such a diagnosis must be carefully considered. Echocardiography can also help to diagnose infective endocarditis in patients with symptoms such as jaundice, abdominal pain, muscle pain, and high fever who do not respond to antibiotic therapy.

## Author Contributions

**Ramtin Rezaie Kalantari:** conceptualization, methodology, project administration, validation. **AmirAbas Sadrian:** conceptualization, investigation, methodology. **Torkan Banaee Rezaeie:** conceptualization, supervision, validation. **Masoud Mortezazadeh:** project administration, validation, visualization, writing – original draft, writing – review and editing. **Dalip Kumar:** methodology, resources, supervision, validation, visualization. **Michael Acidri:** methodology, supervision, writing – review and editing. **Abbas Mofidi:** formal analysis, project administration, supervision, validation.

## Ethics Statement

In this study, no additional costs and procedures were imposed on the patient. We reported the retrograde standard treatment process of the patient. We maintained the patient's privacy, and his written consent was obtained.

## Consent

The patient has consented to the participation and publication of this case report.

## Conflicts of Interest

The authors declare no conflicts of interest.

## Data Availability

The data that support the findings of this study are available from the corresponding author (M.M.) upon reasonable request.
